# A case of mucopolysaccharidosis I with severely increased albuminuria and Graves’ disease, and functional analysis of previously uncharacterized variant

**DOI:** 10.1186/s13023-026-04527-8

**Published:** 2026-08-07

**Authors:** Kun Dong, Junran J. Peng, Xue Zhou, Qiusha Zhu, Changhong Li, Yan Yang

**Affiliations:** 1 Division of Endocrinology, Department of Internal Medicine, Tongji Hospital, Tongji Medical College, Huazhong University of Science and Technology, Wuhan, Hubei China; 2 Hubei Clinical Medical Research Center for Endocrinology and Metabolic Diseases, Wuhan, Hubei China; 3 Branch of National Clinical Research Center for Metabolic Diseases, Wuhan, Hubei China; 4 Nanjing AscendRare Pharmaceutical Technology Co. Ltd., Nanjing, Jiangsu China

**Keywords:** Mucopolysaccharidosis I (MPS I), α-L-iduronidase (*IDUA*) gene, Lysosomal storage disorders, Functional variant analysis, Severely increased albuminuria, Graves’ disease

## Abstract

**Background:**

Mucopolysaccharidoses (MPS) are a group of inherited lysosomal storage disorders which results in accumulation of glycosaminoglycans throughout the body. MPS I is a subtype characterized by mutations in the α-L-iduronidase (IDUA) gene with subsequent multiorgan dysfunction.

**Case description:**

Here, we describe an adult patient with MPS I who presented with anasarca, as well as severely increased albuminuria, and was initially diagnosed with hyperthyroidism. Subsequent testing revealed clinical characteristics and deficient IDUA enzyme activity consistent with MPS I. Genetic testing identified two variants of interest in the IDUA gene: IDUA c·265C > T (p.R89W) and IDUA c.1403-2A > G.

**Methods:**

IDUA enzymatic activity was measured in HEK293T cells transfected with wildtype or p. R89W mutant IDUA expression constructs using the fluorogenic substrate 4-methylumbelliferyl-α-L-iduronide (4MU-iduronide). A minigene splicing assay was performed using IDUA exon 9–11 (including intron 9 and 10) DNA fragments cloned into the pMini-CopGFP vector, with the mutant plasmid containing the IDUA c.1403-2A > G variant generated using site-directed mutagenesis. HEK293T cells were transfected with either the wildtype or mutant plasmids, and splice products were separated by gel electrophoresis with distinct bands excised for Sanger sequencing.

**Results:**

The IDUA c·265C > T (p.R89W) mutant construct was cloned into HEK293T cells and showed significantly decreased intracellular (10.86 ± 0.36%, *p* < 0.01) and extracellular (1.18 ± 0.09%, *p* < 0.01) enzymatic activity compared to wild type. A minigene splicing assay of the previously uncharacterized IDUA c.1403-2A > G splice site variant found retention of intron 9 between exon 9 and 10 leading to a premature termination codon.

**Conclusion:**

This manuscript describes a unique clinical presentation of MPS I with simultaneous Graves’ disease and severely increased albuminuria. Functional analysis of the IDUA variants identified in our patient adds to the current understanding of genetic variants implicated in the disease.

**Supplementary information:**

The online version contains supplementary material available at https://doi.org/10.1186/s13023-026-04527-8.

## Background

Mucopolysaccharidoses (MPS) are a group of rare inherited lysosomal storage disorders characterized by mutations leading to reduced enzymatic degradation of glycosaminoglycans (GAGs). This leads to an accumulation of GAGs which results in multiorgan dysfunction. There are multiple subtypes of MPS, including MPS I, II, III, IV, VI, VII, IX and X. Specifically, MPS I is caused by biallelic pathogenic mutations in the α-L-iduronidase (*IDUA*) gene leading to deficient activity of the IDUA lysosomal enzyme. This leads to an accumulation of heparan sulfates and dermatan sulfates leading to cellular damage [1]. Common clinical presentations for MPS I patients include cognitive impairment, skeletal dysplasia, cardiac valvular diseases, hepatosplenomegaly, ocular abnormalities and hearing loss [2]. The severity of MPS I differs based on the mutations involved, with complete loss-of-function mutations leading to a more severe phenotype called Hurler syndrome, while hypomorphic mutations lead to a more attenuated phenotype called Scheie syndrome. There is also Hurler-Scheie syndrome which is an intermediate phenotype. However, as with many developmental disorders, the actual distribution of phenotypes associated with MPS I is on a spectrum rather than categorical owing to the vast number of genetic variants that are implicated in the disease [3].

Here, we present a unique case of a patient with MPS I who initially presented to Tongji Hospital in Wuhan, China with anasarca, thought to be secondary to hyperthyroidism. However, subsequent investigations revealed features in-keeping with MPS I. This was further supported with genetic testing, which identified compound heterozygous variants of interest in the *IDUA* gene. Subsequent functional testing was done to demonstrate the deleterious effects of these variants.

## Case description

A 27-year-old female born to non-consanguineous parents presented to Tongji Hospital in Wuhan, China in September, 2025 with a 3-month history of bilateral lower extremity edema. Over the same time frame, she reported heat intolerance, excessive sweating and polyphagia. Blood work done 3 months prior to her presentation showed overt hyperthyroidism with a thyroid-stimulating hormone (TSH) level of 0.005 mU/L (reference range 0.27–4.20 mU/L), free thyroxine (fT4) level of 55.3 pmol/L (reference range 12.0–22.0 pmol/L), free triiodothyronine (fT3) level of 17.4 pmol/L (reference range 3.1–6.8 pmol/L), TSH receptor antibody (TRAb) of 26.2 U/L (reference range 0–1.58 U/L), and TSH receptor-stimulating antibody (TSAb) of 2.3 U/L (reference range < 0.1 U/L). Thyroid ultrasound performed at a community hospital found a heterogenous and enlarged thyroid gland. At this point, she was started on methimazole 10 mg three times daily with slight improvement in her symptoms.

One month after initiation of methimazole, the patient developed hypothyroidism while on methimazole. Therefore, her methimazole dose was decreased to 5 mg daily. Two weeks prior to her presentation to Tongji Hospital, the patient developed anasarca with worsening of her lower extremity edema along with neck swelling, facial edema, abdominal distension, orthopnea and shortness of breath on exertion. Given these symptoms, she decided to discontinue her methimazole on her own.

On presentation to Tongji Hospital, physical examination revealed tachycardia and a blood pressure of 104/80 mmHg. Her height was 165 cm, weight was 72 kg, and body mass index (BMI) was 26.4. She was noted to have course facial features with facial edema. On cardiac examination, a holosystolic murmur was auscultated at the apex radiating to the left axilla. Her abdomen was distended but non-tender with no hepatosplenomegaly. There was bilateral 4+ edema to her lower extremities. Further examination revealed mild right and moderate left conductive hearing loss.

Initial lab work revealed mild anemia, mildly elevated cholestatic liver enzymes and bilirubin, and normal creatinine. High-sensitivity cardiac troponin was mildly elevated at 55.1 pg/mL and N-terminal pro-B-type natriuretic peptide (NT-proBNP) was elevated at 4143 pg/mL. Her urinalysis was quite active with 3+ proteins detected in addition to trace blood, leukocyte esterase, ketones and bilirubin. Thyroid function testing revealed a TSH of 0.302 uIU/mL (reference range 0.27–4.2 uIU/mL), fT4 of 16.52 ng/L (reference range 9.26–16.80 ng/L) and fT3 of 3.07 pg/mL (reference range 2.0–4.4 pg/mL).

A pan-CT done on admission found small bilateral pleural effusions, a small pericardial effusion, and ascites. Acetabular and femoral head dysplasia were also noted (Supplemental Figure S1). On CT head, an arachnoid cyst was seen within an anteroposteriorly enlarged sella turcica. A cardiac MRI done revealed cardiomegaly with decreased biventricular systolic function (left ventricular ejection fraction of 15%) and hypertrabeculation. There was also endocardial fibrosis within the interventricular septum. Multiple valvular abnormalities were noted, including mildly thickened mitral, tricuspid and aortic valves with associated aortic stenosis and insufficiency, mitral insufficiency and tricuspid insufficiency.

**Fig. 1:**
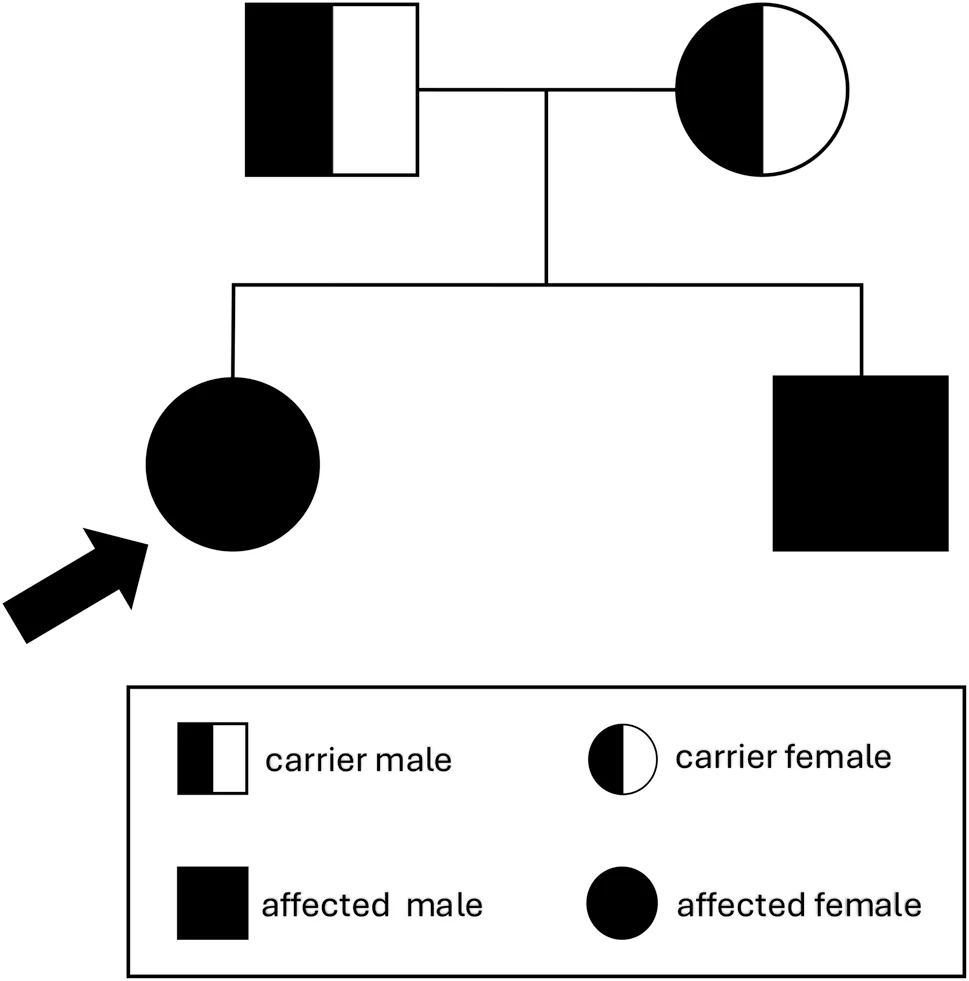
Pedigree of the patient’s family. Arrow points to the patient (ie. The proband). Carrier individuals carry a single pathogenic *IDUA* variant (either *IDUA* c.265C > T (p.R89W) or *IDUA* c.1403-2A > G), while affected individuals carry both variants and exhibit the MPS I phenotype

We subsequently sent the patient’s blood to test for leukocyte IDUA enzyme activity, which was found to be deficient at 0.1 nmol/(1 h·mg) (reference range > 17 nmol/(1 h·mg)), supporting a biochemical diagnosis of MPS I. One month after the patient’s initial presentation, whole-exome sequencing was performed and identified two variants of interest suspected to cause MPS I: *IDUA* c.265C > T (p.R89W) is a known pathogenic missense variant in exon 2 of the *IDUA* gene inherited from her father, while *IDUA* c.1403-2A > G is a previously uncharacterized splice acceptor variant in intron 9 of the *IDUA* gene inherited from her mother (Fig. 1, Supplemental Figure S2). Genetic testing of the patient’s brother yielded the same variants.

Interestingly, while the patient was asymptomatic at birth, she started developing coarse facial features and diffuse joint disease in her childhood and was suspected to have MPS previously. Her brother was also noted to have similar clinical characteristics as a child, though he did not develop heart failure secondary to valvular disease as in our patient. Unfortunately, no additional investigations were done nor formal diagnosis made prior to this admission largely due to the family’s financial constraints.

For management, the patient was restarted on methimazole 2.5 mg daily. Furosemide was started for diuresis, spironolactone for heart failure with reduced ejection fraction (HFrEF) goal-directed medical therapy (GDMT) and ivabradine for heart rate management. At discharge, the patient’s weight was 65 kg, down approximately 7 kg compared to admission, with significant improvement in her anasarca. On outpatient follow-up, the patient’s dry weight was measured at 61 kg. She was additionally started on bisoprolol and dapagliflozin for HFrEF GDMT.

Despite her valvular abnormalities, the patient was not planning on pursuing surgical management at the time this report was written. She also opted not to proceed with enzyme replacement therapy for management of her MPS I.

## Methods

### Expression vector construction and site-directed mutagenesis

Full-length human *IDUA* cDNA (RefSeq NM_000203.5) was cloned into the pcDNA3.1 eukaryotic expression vector to generate the wild-type construct (pcDNA3.1-IDUA-WT). The missense mutation *IDUA* c.265C > T (p.R89W) was introduced into the WT template to create the mutant construct (pcDNA3.1-IDUA-R89W). Both constructs were confirmed by sequencing.

### IDUA enzymatic activity assay

HEK293T cells were transfected with pcDNA3.1-IDUA-WT, pcDNA3.1-IDUA-R89W, or pcDNA3.1 (empty vector negative control) using Lipomaster 3000 Transfection Reagent (Vazyme, China). Forty-eight hours after transfection, both cell lysates and culture supernatants were collected. Cells were homogenized in lysis buffer containing 0.1% Triton X-100 and centrifuged to obtain the supernatant. Protein concentration was determined using the Bradford protein assay (Coomassie Brilliant Blue G-250 dye-binding method).

IDUA enzymatic activity was measured using the fluorogenic substrate 4-methylumbelliferyl-α-L-iduronide (4MU-iduronide). Briefly, cell lysate or cell culture supernatant (25 µL) was incubated with 25 μL of substrate (diluted in 0.4 mol/L sodium formate buffer, pH 3.5) at 37 °C for 15 minutes. The reaction was terminated by adding 200 μL of glycine-carbonate buffer (pH 10.4). Fluorescence was measured using a microplate reader (excitation 355 nm; emission 460 nm). Enzyme activity was calculated based on a 4-methylumbelliferone (4-MU) standard curve and normalized to protein amount (nmol/15 min/µg protein). The enzymatic activity was measured at a final saturating substrate concentration of 2 mM. All enzymatic activity assays were performed in technical triplicates (*n* = 3 technical replicates from the transfected cell preparations).

### Minigene splicing assay

To investigate the effect of the intron variant *IDUA* c.1403-2A > G on pre-mRNA splicing, a minigene assay was performed. Genomic DNA fragments spanning *IDUA* exon 9 to exon 11 (including intron 9 and intron 10) were amplified and cloned into the pMini-CopGFP vector. The mutant plasmid containing the *IDUA* c.1403-2A > G variant was generated using site-directed mutagenesis. The wild-type (WT) and mutant (MT) plasmids were verified by Sanger sequencing.

HEK293T cells were cultured in DMEM supplemented with 10% fetal bovine serum (FBS). Cells were transiently transfected with either the WT or mutant minigene plasmids using Lipomaster 3000 Transfection Reagent (Vazyme, China) according to the manufacturer’s instructions. 48 hours post-transfection, total RNA was extracted, and cDNA was synthesized. Splicing patterns were analyzed by RT-PCR using vector-specific primers (MiniRT-F: 5’-GGCTAACTAGAGAACCCACTGCTTA-3’ and MiniRT-R: 5’-GTTTAAACGGGCCCTCTAGACTCGA-3’). PCR products were separated by agarose gel electrophoresis, and distinct bands were excised for Sanger sequencing to determine the precise splicing events.

## Results

We identified two genetic variants of interest in our patient suspected to cause MPS I. The *IDUA* c.265C > T (p.R89W) missense variant in exon 2 of the *IDUA* gene inherited from the patient’s father is known to be pathogenic. The second variant is a previously uncharacterized splice acceptor variant *IDUA* c.1403-2A > G in intron 9 of the *IDUA* gene inherited from the patient’s mother (Fig. 1).

To assess the functional impact of the p.R89W variant, we measured IDUA enzymatic activity in HEK293T cells expressing wild-type protein, p.R89W mutant protein, and an empty vector negative control (Table 1). In the intracellular fraction, the p.R89W mutant exhibited significantly reduced enzymatic activity, approximately 10.9% of the wild-type transfected cells (10.86 ± 0.36% of wild type, *p* < 0.01) at a 4MU-iduronide substrate concentration of 2 mM. The activity of the empty vector control was negligible at 0.22 ± 0.02% of the wild-type cells (*p* < 0.01), representing HEK293T endogenous IDUA activity.

**Table 1:** Mean intracellular and extracellular α-L-iduronidase (IDUA) enzymatic activity of HEK293T cells transfected with the p.R89W mutant construct (pcDNA3.1-IDUA-R89W) and empty vector negative control (pcDNA3.1) expressed as a percentage of the wild type IDUA enzymatic activity (pcDNA3.1-IDUA-WT)

	Enzymatic Activity (mean ± SD)
**Intracellular**	
Wild type	100%
Empty vector	0.22 ± 0.02%*
p.R89W mutant	10.86 ± 0.36%*^†^
**Extracellular**	
Wild type	100%
Empty vector	0.99 ± 0.06%*
p.R89W mutant	1.18 ± 0.09%*

All enzymatic activity assays were performed in technical triplicates (*n*=3)

*Indicates statistically significant difference in enzymatic activity compared to wild type

^†^Indicates statistically significant difference in enzymatic activity compared to empty vector negative control

We also analyzed the enzymatic activity in the culture medium to evaluate protein secretion (Table 1). While wild-type IDUA was efficiently secreted and detectable in the supernatant, the extracellular activity of the p.R89W mutant was nearly undetectable (1.18 ± 0.09% of wild type, *p* < 0.01), comparable to the empty vector control (0.99 ± 0.06% of wild type, *p* < 0.01).

To determine the pathogenicity of the splice-site variant *IDUA* c.1403-2A > G, we performed a minigene splicing assay containing exon 9 to exon 11 of the *IDUA* gene. RT-PCR analysis revealed that the wild-type plasmid produced a single expected band of approximately 600 bp, corresponding to the normally spliced product. In contrast, the *IDUA* c.1403-2A > G mutant plasmid produced a larger transcript, suggesting defective splicing. No additional bands corresponding to the normally spliced ~600 bp product were detected in the mutant lanes at visible levels on agarose gels from multiple independent transfections (Supplemental Figure S3).

Sanger sequencing of the RT-PCR products confirmed that the wild-type transcript showed normal exon-exon joining while the mutant transcript exhibited retention of the entire intron 9 sequence. Sequence analysis indicates that this intron retention introduces an in-frame premature termination codon within the retained intron sequence, which is designated as p.G468ext*4 (or p.Gly468_Intron9term) according to the HGVS nomenclature. This modification is predicted to trigger nonsense-mediated mRNA decay (NMD) or lead to a truncated, non-functional protein missing essential catalytic domains.

## Discussion

Mucopolysaccharidoses are a group of lysosomal storage diseases caused by genetic mutations leading to abnormal degradation of glycosaminoglycans [1]. There are multiple subtypes of this disease (MPS I, II, III, IV, VI, VII, IX, and X) with various clinical manifestations and degrees of severity. MPS I is associated with accumulation of heparan sulfate and dermatan sulfate leading to possible disease involving the bones, soft tissue, liver, spleen, heart, lungs, brain and eyes. Classically, there have been three identified subtypes of MPS I: Hurler syndrome (severe phenotype), Hurler/Scheie syndrome (intermediate phenotype) and Scheie syndrome (mild phenotype). However, the most updated understanding of this disease now recognizes the phenotype as on a spectrum rather than categorical [1].

In our patient, we identified two variants of interest in the *IDUA* gene suspected to cause MPS I: the recognized pathogenic *IDUA* c.265C > T (p.R89W) variant and the *IDUA* c.1403-2A > G splice acceptor variant which has not been previously characterized (Table 1).

The p.R89W variant has been previously described in the literature to be associated with the attenuated Scheie phenotype. Bunge et al. (1998) and Hein et al. (2003) each described a patient with this mutation, the first with a p.R89W/p.Q70X genotype and Scheie phenotype and the second with a p.R89W/p.P533R genotype and Scheie phenotype [4, 5]. Fibroblasts from these patients were studied to determine protein quantity and function. Interestingly, while the protein substrate affinity, max rate of enzyme-catalyzed reaction and catalytic efficiency were all multiple folds lower than the wild type cell line, the protein quantity from the mutant cell lines was actually the same or even higher than wild type [4, 5]. In comparison, another patient with a p.Q70X/p.Q70X genotype had a Hurler phenotype and undetectably low protein quantity and reaction rate [4]. This shows that the p.Q70X variant results in almost complete loss of protein function. Similarly, the p.P533R variant when combined with a second severe variant has been noted to result in a Hurler clinical phenotype [5]. Therefore, it appears that the p.R89W mutation preserves enough enzymatic activity to induce the attenuated Scheie phenotype rather than the more severe Hurler phenotype.

In our study, we assessed the effect the p.R89W mutation on IDUA enzyme activity. Similar to previous studies [4, 5], we found that intracellular activity, while significantly decreased at 10.9% of WT, was preserved well above negative control thresholds. Our findings independently cross-validate their observations that the p.R89W alteration permits partial retention of intracellular enzymatic function, explaining the attenuated phenotype. Interestingly, extracellular activity of the p.R89W mutant was comparable to negative controls. While this could point to potential abnormalities in protein folding, processing, and trafficking leading to intracellular retention, it could alternatively reflect lower baseline expression, extracellular protein instability, or decreased catalytic efficiency. Further evaluation via protein-expression profiling and localization data is required to fully elucidate this mechanism and confirm whether chaperone-based therapies could offer clinical utility.

The second variant (*IDUA* c.1403-2A > G) identified in our patient is an intronic splice acceptor site mutation swapping adenosine for guanine. There is one report of this exact mutation on GnomAD in a European (non-Finnish) XX individual, while there is another report of an adenosine for cytosine mutation in the same position in a European (non-Finnish) XY individual [6]. There are no entries for variants in this position on ClinVar [7] and to our best knowledge, there are no associated publications at the time this manuscript was written.

We assessed the *IDUA* c.1403-2A > G variant using a minigene splicing assay. By utilizing a multi-exon minigene spanning exons 9 through 11, we successfully preserved the local genomic sequence context of the variant, minimizing minor artifacts commonly associated with artificial single-exon trapping systems, though it lacks the full endogenous chromatin and transcriptional regulation of the native locus. We found that compared to the wildtype plasmid, the mutant plasmid with this splice-site variant led to a larger transcript. Sequencing of the mutant transcript found retention of the entire intron 9 sequence, which indicates that this mutation disrupts the 3’ splice acceptor site of intron 9. Further sequence analysis indicates that aberrant splicing at this location introduces a premature termination codon within the intron, which is predicted to trigger nonsense-mediated mRNA decay or lead to a truncated, non-functional protein. While our minigene assay demonstrates clear aberrant splicing caused by the IDUA c.1403-2A > G variant, validation using patient-derived RNA or additional independent cell lines would further confirm these findings in a native chromatin and transcriptional context. Additionally, it would aid our understanding to, in the future, perform protein functional studies on this presumed truncated protein to prove lack of enzymatic activity.

Clinically, our patient had significant cardiovascular disease which likely led to her presentation. An inpatient cardiac MRI found evidence of decreased biventricular systolic function (LVEF 15%), increased trabeculation of both ventricles, moderate aortic stenosis, mild-to-moderate aortic regurgitation, mild mitral stenosis, mild-to-moderate mitral regurgitation and severe tricuspid regurgitation. As with our current understanding of the disease, cardiac valvular disease is a common manifestation of MPS I suspected to be due to progressive GAG infiltration of the valvular tissue [8]. However, while most MPS I patient have cardiac manifestations, increased trabeculation has not been described in the literature as associated with the disease. It is unclear to us at this point whether this finding is a direct manifestation of MPS I or if this is perhaps part of the myocardial remodeling process in systolic dysfunction [9].

Our patient also had 3+ proteinuria on urinalysis with a urine albumin-to-creatinine ratio of 713.5 ug/mg, but normal serum creatinine and serum albumin levels. Interestingly, while MPS is known to have a broad spectrum of clinical manifestations and multi-organ involvement, renal involvement is not commonly seen except in MPS plus syndrome (MPSPS). MPSPS is caused by a homozygous mutation in the *VPS33B* gene and is characterized by typical MPS features with additional clinical characteristics including congenital heart defects, hematopoietic system disorders and renal dysfunction [10]. Notably, most patients had various degrees of proteinuria with ultrasonography showing nephromegaly and increased echogenicity. Renal biopsies showed focal segmental glomerulosclerosis, periglomerular fibrosis and inflammatory cell infiltration [10].

It is difficult to understand the exact reason for proteinuria in our patient with MPS I as a renal biopsy was not done. However, Taylor et al. (1986) reported two cases of nephrotic syndrome in patients with MPS I on whom they performed postmortem renal tissue sampling. Based on their publication, both patients had the Hurler phenotype and were diagnosed with MPS I before the age of 2 [11]. Both patients were then noted to have hypertension later in their lives with elevated peripheral renin levels. Both patients had a history of being admitted to hospital with edema, elevated 24-hour protein excretion, elevated creatinine levels and hypoalbuminemia. Postmortem examination of tissue from the kidneys of these patients revealed swollen, vacuolated visceral epithelial cells and enlarged podocytes with the presence of acid mucopolysaccharides on special stains [11].

Based on these tissue findings, Taylor et al. (1986) postulated that the accumulation of abnormal heparin sulfate-containing metabolites in the glomerular basement membranes distorts the charge-selective barrier and increases permeability to negatively charged molecules, including proteins. Interestingly, two additional patients from their hospital had similar postmortem renal tissue findings without clinical signs of nephrotic syndrome [11]. Therefore, it appears that renal involvement exists on a spectrum, similar to other manifestations of MPS I. In our patient, the exact etiology of her albuminuria remains unclear, but FSGS secondary to MPS is a possibility. Further investigation is warranted to assess for additional correlation between MPS and renal disease and further elucidate the underlying pathophysiology.

Prior to her presentation to hospital, our patient also had hyperthyroidism requiring methimazole treatment. Based on current understanding of MPS I or other mucopolysaccharidoses, thyroid involvement is not known to be a common manifestation. There is limited research on how MPS affects the thyroid. Previous autopsy results from MPS II patients found cytoplasmic vacuolar changes in the follicular epithelial cells of the thyroid gland [12, 13]. However, a case series out of Turkey containing 25 patients with MPS I, II, III, IVA, and VI found all patients had normal thyroid hormone levels [14]. Ultrasonography of the thyroid gland was also done, which revealed only 2 patients had slightly heterogenous echotexture and one patient had slightly increased vascularization on colour Doppler. Therefore, it was concluded that MPS is not usually associated with any thyroid dysfunction or sonographic abnormalities [14]. Interestingly, there is one case report from Indonesia of a patient with MPS II and hyperthyroidism on PTU [15]. However, the etiology of the hyperthyroidism was not further explored in the publication and therefore, it is difficult to ascertain whether the altered thyroid function is associated with MPS or if it is in fact a separate diagnosis. In our patient, a thyroid biopsy was not done and therefore, it is unclear whether there exists GAG accumulation in the thyroid tissue contributing to her presentation. However, based on her positive TSH-receptor antibody titre, we suspect Graves’ disease as the primary etiology.

## Conclusion

In this manuscript, we describe a unique presentation of an adult female patient with suspected MPS I caused by compound heterozygous mutations in the *IDUA* gene. One of the variants is a recognized pathogenic missense variant which results in decreased IDUA enzymatic function. The second variant is a previously uncharacterized loss-of-function splice acceptor site mutation which causes retention of an intron leading to a premature termination codon. Our 27-year-old patient presented with many of the typical MPS I clinical manifestations, including heart failure with significant valvular abnormalities, coarse facial features, classic bone involvement, and hearing loss. Interestingly, our patient did also have severely increased albuminuria and Graves’ disease, which are not known to be associated with MPS I. As such, these findings provide areas for further observation and investigation.

## Electronic supplementary material

Below is the link to the electronic supplementary material.

Supplementary Material 1

## Data Availability

Requests to access the datasets should be directed to the corresponding author.

## Abbreviations

MPS: Mucopolysaccharidosis
IDUA: α-L-iduronidase
MPSPS: MPS plus syndrome
TSH: Thyroid-stimulating
fT4: Free thyroxine
fT3: Free triiodothyronine
TRAb: TSH receptor antibody
TSAb: TSH receptor-stimulating antibody
NT-proBNP: N-terminal pro-B-type natriuretic peptide
HFrEF: Heart failure with reduced ejection fraction
4MU-iduronide: 4-methylumbelliferyl-α-L-iduronide
