# Supplementary material for: A case of mucopolysaccharidosis I with severely increased albuminuria and Graves’ disease, and functional analysis of previously uncharacterized variant
Source: Orphanet J Rare Dis. 2026 Aug 7;21:399. doi: 10.1186/s13023-026-04527-8 (PMC13741428; doi:10.1186/s13023-026-04527-8)
Supplement: Supplementary file 1 [file 13023_2026_4527_MOESM1_ESM.docx]

**Supplemental Figure S1.** Computed tomography (CT) images of the pelvis demonstrating acetabular and femoral head dysplasia. (a) Coronal view. (b) Axial view.

**Supplemental Figure S2.** Sanger sequencing chromatograms of the *IDUA* c.265C>T (p.R89W) and *IDUA* c.1403-2A>G variants in the proband, mother and father.

**Supplemental Figure S3.** The uncropped agarose gel showing RT-PCR products from the Minigene Splicing Assay. (A) shows the pMini-CopGFP-IDUA pre-mRNA sequence containing exon 9-11, including intron 9 and 10, before RNA splicing occurs. (B) shows the splice product of the pMini-CopGFP-IDUA-WT wild-type plasmid. (C) shows the splice product of the pMini-CopGFP-IDUA-MT *IDUA* c.1403-2A>G mutant plasmid. A DNA ladder (100-1000 bp) is shown on the left for size reference with the band indicating 600 bp marked by an arrow.
